# Drug-induced caspase 8 upregulation sensitises cisplatin-resistant ovarian carcinoma cells to rhTRAIL-induced apoptosis

**DOI:** 10.1038/bjc.2011.84

**Published:** 2011-04-12

**Authors:** E W Duiker, A Meijer, A R M van der Bilt, G J Meersma, N Kooi, A G J van der Zee, E G de Vries, S de Jong

**Affiliations:** 1 Department of Medical Oncology, University Medical Centre Groningen, University of Groningen, Hanzeplein 1, Groningen 9713 GZ, The Netherlands; 2 Department of Gynaecologic Oncology, University Medical Centre Groningen, University of Groningen, Hanzeplein 1, Groningen 9713 GZ, The Netherlands

**Keywords:** TRAIL, apoptosis, caspase 8, DR5, p53, cisplatin

## Abstract

**Background:**

Drug resistance is a major problem in ovarian cancer. Triggering apoptosis using death ligands such as tumour necrosis factor-related apoptosis inducing ligand (TRAIL) might overcome chemoresistance.

**Methods:**

We investigated whether acquired cisplatin resistance affects sensitivity to recombinant human (rh) TRAIL alone or in combination with cisplatin in an ovarian cancer cell line model consisting of A2780 and its cisplatin-resistant subline CP70.

**Results:**

Combining cisplatin and rhTRAIL strongly enhanced apoptosis in both cell lines. CP70 expressed less caspase 8 protein, whereas mRNA levels were similar compared with A2780. Pre-exposure of particularly CP70 to cisplatin resulted in strongly elevated caspase 8 protein and mRNA levels. Caspase 8 mRNA turnover and protein stability in the presence or absence of cisplatin did not differ between both cell lines. Cisplatin-induced caspase 8 protein levels were essential for the rhTRAIL-sensitising effect as demonstrated using caspase 8 small-interfering RNA (siRNA) and caspase-8 overexpressing constructs. Cellular FLICE-inhibitory protein (c-FLIP) and p53 siRNA experiments showed that neither an altered caspase 8/c-FLIP ratio nor a p53-dependent increase in DR5 membrane expression following cisplatin were involved in rhTRAIL sensitisation.

**Conclusion:**

Cisplatin enhances rhTRAIL-induced apoptosis in cisplatin-resistant ovarian cancer cells, and induction of caspase 8 protein expression is the key factor of rhTRAIL sensitisation.

In ovarian cancer, the majority of tumours acquire drug resistance. Response rates to first-line platinum-based therapy are more than 80%, but most patients with advanced disease will finally relapse and die because of acquired drug resistance (Agarwal and Kaye, 2003). Chemoresistance is attributed to numerous mechanisms, which can be broadly divided into decreased DNA damage response via p53 and increased cell survival, mainly through defects in apoptosis (Wernyj and Morin, 2004). A meta-analysis showed that aberrant p53 status results in a worse 5-year survival for ovarian cancer patients (Crijns *et al*, 2003). Triggering apoptosis directly via the extrinsic apoptotic pathway might circumvent escape mechanisms developed by cancer cells. Especially the recombinant human (rh) form of the death ligand tumour necrosis factor-related apoptosis inducing ligand (TRAIL) is considered to be interesting for clinical use because of its ability to induce apoptosis in several types of cancer cell lines and xenografts (Wiley *et al*, 1995; Pitti *et al*, 1996; Ashkenazi *et al*, 2008). Preliminary data from a phase I trial with rhTRAIL showed no major toxicity (Herbst *et al*, 2010). TRAIL induces apoptosis by binding to death receptor 4 (DR4) and DR5 (Ashkenazi *et al*, 2008). Binding to these DRs causes receptor trimerisation and recruitment of the adaptor protein Fas-associated death domain (FADD). This in turn recruits caspase 8, resulting in the formation of the death-inducing signalling complex (DISC) (Peter, 2000). Binding of caspase 8 to the DISC causes its activation (Boatright *et al*, 2003), with subsequent activation of effector caspases 3, 6 and 7, which will execute apoptosis. The cellular FLICE-inhibitory protein (c-FLIP), which is vastly homologous to caspase 8 but lacks enzymatic activity, can also associate with the DISC, blocking activation of caspase 8 through competition for binding sites. It has also been stated that c-FLIP may function as an activator of caspase 8 under specific circumstances (Micheau *et al*, 2002; Boatright *et al*, 2004). In addition, the intrinsic apoptotic pathway can be activated by caspase 8 through cleavage of the BH3-only protein Bid that triggers perturbation of the mitochondria by Bax and Bak and finally activation of caspase 9 and effector caspases (Li *et al*, 1998). Numerous studies have demonstrated that drug resistance in cancer cells including ovarian cancer cells, could be prevented or overcome by combining rhTRAIL with chemotherapeutics (Mahalingam *et al*, 2009). In a previous study, we showed that cisplatin can sensitise ovarian cancer cells to rhTRAIL-induced apoptosis *in vitro* as well as in a bioluminescent ovarian cancer xenograft model (Duiker *et al*, 2009). The described mechanisms involved in modulation in ovarian cancer cells were established by comparison of cell lines with different background and sensitivity patterns, which impede establishment of causal relationships. Therefore, we investigated in an isogenic model of cisplatin resistance the molecular determinants for rhTRAIL sensitivity, the mechanism of synergy between cisplatin and rhTRAIL and the role of functional p53 in this synergy.

## Materials and Methods

### Cell lines

The ovarian cancer cell lines A2780 and its five-fold cisplatin-resistant subline CP70, which carry wild-type and functional p53, were a kind gift from Dr Hamilton (Fox Chase Cancer Center, Philadelphia, PA, USA) (Godwin *et al*, 1992). Cisplatin resistance in CP70 can partly be explained by an increased DNA repair mechanism and a higher intracellular glutathione content (Johnson *et al*, 1994). Cells grew as monolayers in RPMI 1640 (Life Technologies Breda, the Netherlands), supplemented with 10% heat-inactivated fetal calf serum (FCS) (Bodinco BV, Alkmaar, the Netherlands) and 0.1 M L-glutamine. Cell lines were cultured in a humidified atmosphere with 5% CO_2_.

### Cytotoxicity assay

The microculture tetrazolium assay was used to measure cytotoxicity as described earlier (Duiker *et al*, 2009). Treatment consisted of continuous incubation with cisplatin (Pharmacochemie BV, Haarlem, the Netherlands) or rhTRAIL (produced as described earlier (Ashkenazi *et al*, 1999)) for 96 h. The mean IC_50_±s.d. was determined in three experiments, each performed in quadruplicate.

### Determination of apoptosis

In 96-well plates, cells were incubated with either rhTRAIL or cisplatin, or both. The cells were exposed to cisplatin for 4 h, washed with phosphate-buffered saline (PBS: 6.4 mM Na_2_HPO_4_; 1.5 mM KH_2_PO_4_; 0.14 mM NaCl; 2.7 mM KCl; pH=7.2) twice and incubated in regular culture medium. At 20 h after administration of cisplatin, rhTRAIL was added for 4 h. Following rhTRAIL treatment, nuclear chromatin was stained with acridine orange to identify apoptosis by fluorescence microscopy.

### SDS–polyacrylamide gel electrophoresis and western blotting

Cells were treated with rhTRAIL and/or cisplatin as described above. Treatment with the proteasome inhibitor MG132 (Calbiochem, Breda, the Netherlands) (0.5 *μ*M) lasted for 24 h and rhTRAIL was added for the last 4 h. The caspase inhibitor I (zVAD) (Calbiochem) was added 2 h before MG132. Cycloheximide (CHX) (Sigma-Aldrich, Zwijndrecht, the Netherlands) exposure was performed as indicated. After treatment, total cell lysates were generated and western blotting was performed as described previously (Duiker *et al*, 2009), using the following primary antibodies: Fas-associated death domain and XIAP (Transduction Laboratories, Alphen a/d Rijn, the Netherlands), Bax, Bak, Bcl-2, Bcl-X_S/L_ and p53-DO-1 (Santa Cruz, Heerhugowaard, the Netherlands), caspase 9 and Cip1/Waf1 (p21) (BD Biosciences, Alphen a/d Rijn, the Netherlands), caspase 8 and cleaved caspase 3 (Cell Signaling, Leusden, the Netherlands). The Bid- and FLIP NF6 antibodies were kindly provided by Dr J Borst (The Netherlands Cancer Institute, Amsterdam, the Netherlands) and Dr ME Peter (The Ben May Institute for Cancer Research, Chicago, IL, USA), respectively. Secondary antibodies conjugated with horseradish peroxidase were obtained from DAKO (Glostrup, Denmark). Equal protein loading was confirmed by *β*-actin (ICN Pharmaceuticals, Zoetermeer, the Netherlands). Visualisation was performed with BM Chemiluminescence Blotting Substrate (POD) or LumiLight Plus Western Blotting Substrate (Roche Diagnostics, Almere, the Netherlands).

### Flow cytometry

Cells were collected using trypsin and washed once with cold PBS. Cells were subsequently washed twice with cold PBS containing 2% FCS and 0.1% sodium azide and incubated with phycoerythrin (PE)-conjugated mouse monoclonal antibodies against DR4, DR5, decoy receptor 1 (DcR1) and decoy receptor 2 (DcR2). Mouse PE-labelled IgG1 and IgG2B were used as isotype controls. All PE-labeled antibodies were purchased from R&D Systems (Oxon, UK). Receptor membrane expression was analysed using a flow cytometer (Epics Elite, Coulter Electronics, Hialeah, FL, USA) and is shown as mean fluorescent intensity (MFI) of all analysed cells.

### RNA interference and gene transfection

Small-interfering RNAs (siRNAs) were synthesised by Eurogentec (Seraing, Belgium). The double-stranded c-FLIP siRNA was 5′-GAGGUAAGCUGUCUGUCGG-dTdT-3′ (sense) and 5′-CCGACAGACAGCUUACCUC-dTdT-3′ (antisense). P53 siRNA 5′-GCAUGAACCGGAGGCCCAU-dTdT-3′ (sense) and 5′-AUGGGCCUCCGGUUCAUGC-dTdT-3′ (anti-sense) was designed according to Martinez *et al* (2002). Oligonucleotides specific for luciferase mRNA served as a negative control (Elbashir *et al*, 2001). Caspase 8 siRNA 5′-CUACCAGAAAGGUAUACCU-dTdT-3′ (sense) and 5′-AGGUAUACCUUUCUGGUAG-dTdT-3′ (anti-sense) was designed according to Chun *et al* (2002) and negative control siRNA from Eurogentec was used. Subconfluent cells were incubated in unsupplemented Optimem medium and transfected with siRNA (up to 133 nM) using Oligofectamine reagent according to the manufacturer's protocol (Invitrogen, Breda, the Netherlands). For the caspase 8 overexpression experiments, cells were transfected with pcDNA3-FLICE (a gift from J Borst) using Fugene. The next day, cells were treated with cisplatin and/or rhTRAIL as described above, collected and used for protein isolation, cytospins, FACS experiments and/or an apoptosis assay. Cytospins were immunohistochemically stained for caspase 8 as described earlier (Duiker *et al*, 2010).

### Real-time RT–PCR

Total RNA was isolated by guanidine isothiocyanate–phenol–chloroform extraction with TRIzol (Invitrogen) and purified using the RNeasy mini Kit and on-column DNase I digestion (Qiagen, Leusden, the Netherlands) according to the manufacturer's instructions. Complementary DNA was synthesised from 1600 ng purified RNA as described by the manufacturer's protocol (Life Technologies) using oligo (dT)11 primers and MMLV transcriptase. Real-time RT–PCR was performed in 96-wells plates on a MyiQ real-time detection system (Bio-Rad, Veenendaal, the Netherlands) with GAPDH as a housekeeping reference gene. Primer sequences of caspase 8 were 5′-GGAGCTGCTCTTCCGAATTA-3′ (forward) and 5′-GCAGGTTCATGTCATCATCC-3′ (reverse) and those of GAPDH were 5′-CACCACCATGGAGAAGGCTGG-3′ (forward) and 5′-CCAAAGTTGTCATGGATGACC-3′ (reverse). Amplification of the samples in triplicate was carried out in a final reaction volume of 25 *μ*l, containing 1 × iQ SYBR Green Supermix (Bio-Rad), 5 *μ*M of each primer and 5 *μ*l cDNA (1 : 50). The thermocycling programme used for each real-time RT–PCR consisted of an initial 3-min denaturation at 95 °C, followed by 40 cycles of 15-s denaturation at 95 °C, 20 s primer annealing at the primer specific *T*_ann_ and 30-s fragment elongation at 72 °C. A melting curve was obtained at the end of each 40 cycles of amplification to determine the presence of a unique reaction product. To determine RT–PCR efficiency and initial starting quantity of the samples, a standard curve was generated using samples from a 1 : 3 dilution series of total starting cDNA.

### Statistical analysis

All experiments were performed at least three times on different occasions. Analysis included double-sided non-paired Student's *t*-test. A *P*-value <0.05 was considered significant.

## Results

### Combination of cisplatin and rhTRAIL causes enhanced induction of apoptosis

**Figure 1:**
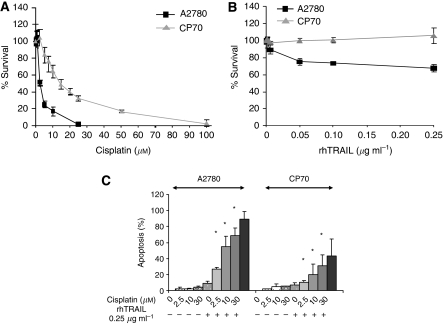
Resistance to cisplatin causes cross-resistance to rhTRAIL. Combination therapy overcomes resistance. (**A**) Survival after 96 h exposure to 0–25 *μ*M (A2780) or 0–100 *μ*M (CP70) cisplatin and (**B**) survival after 96 h exposure to 0 *μ*g ml^−1^–0.25 *μ*g ml^−1^ rhTRAIL as measured by cytotoxicity assays. (**C**) To determine apoptosis induction, cells were treated for 4 h with cisplatin (2.5, 10 and 30 *μ*M) after which cisplatin was washed away. Twenty hours later, the cells were treated for 4 h with 0.25 *μ*g ml^−1^ rhTRAIL. Apoptosis was determined with acridine orange staining. Apoptosis in the combinations marked with ^*^ was significantly enhanced (*P*<0.05) over apoptosis after single-agent treatment. Data represent the mean±s.d. of at least three independent experiments.

A2780 was moderately sensitive to cisplatin treatment for 96 h, with an IC_50_ of 2.6 *μ*M in a survival assay. The subline CP70 was five-fold resistant to cisplatin, with an IC_50_ of 14.7 *μ*M (Figure 1A). The subline CP70 was rhTRAIL resistant, and A2780 was moderately sensitive, as IC_50_ was not reached using up to 0.25 *μ*g ml^−1^ in a survival assay for 96 h (Figure 1B). On the basis of previous data, cells were treated for 4 h with cisplatin, recovered for 20 h and treated with rhTRAIL for 4 h for the apoptosis assay (Duiker *et al*, 2009). Recombinant human TRAIL (0.25 *μ*g ml^−1^) induced moderate levels of apoptosis in A2780, ∼20%, whereas CP70 was not sensitive to rhTRAIL. Combination of cisplatin with rhTRAIL enhanced apoptosis in both cell lines, with ∼80% apoptosis in A2780 and ∼40% apoptosis in CP70 (Figure 1C).

### The resistant cell line CP70 has reduced caspase 8 protein levels

**Figure 2:**
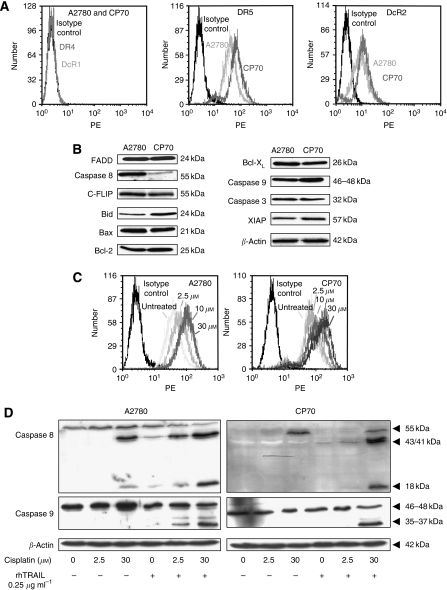
Cellular characteristics involved in sensitivity to rhTRAIL. Cisplatin can induce caspase 8 protein in the low caspase 8-expressing CP70. (**A**) Basic levels of TRAIL receptor membrane expression in A2780 and CP70 as determined by FACS analysis. Receptor expression is expressed as MFI. (**B**) Western blot analysis of basic protein expression levels of key determinants of the TRAIL pathway in A2780 and CP70. (**C**) The effect of cisplatin on DR5 expression in both cell lines. Flow cytometry was performed at 24 h after 4 h incubation with cisplatin. (**D**) Western blot analysis of caspases 8 and 9 after exposure to cisplatin and rhTRAIL. A2780 and CP70 were incubated with 2.5 *μ*M or 30 *μ*M cisplatin during 4 h, after which the cells were washed. Twenty hours later, unexposed or cisplatin-exposed cells were treated during 4 h with 0.25 *μ*g ml^−1^ rhTRAIL. *β*-Actin serves as a loading control. The blots are representative for at least three independent experiments.

To determine which cellular characteristics could account for the different sensitivity patterns to rhTRAIL, key components of the TRAIL signalling pathway were analysed. Membrane expression of DR4 and DcR1 were almost undetectable in both cell lines. Decoy receptor 2 expression was similar, whereas DR5 was increased in CP70 (MFI=120±31) *vs* A2780 (MFI=53±26) (Figure 2A). Western blot analysis showed similar expression of FADD, c-FLIP_L_, c-FLIP_s_, caspases 9 and 3, Bid, Bax, Bak, Bcl-2 and Bcl-X_L_ XIAP levels in A2780 and CP70. Remarkably, caspase 8 levels were lower in CP70 than in A2780 (Figure 2B).

### Cisplatin induces upregulation of caspase 8 protein

Next, we evaluated the effect of cisplatin on TRAIL-receptor expression and the effect of different regimens on cleavage of caspases 8 and 9. Cisplatin did not affect DR4 and DcR1 levels. Decoy receptor 2 was moderately upregulated, but DR5 was strongly induced upon exposure to cisplatin in both cell lines (Figure 2C). In response to cisplatin, moderate activation of caspase 8 was induced in A2780. Recombinant human TRAIL alone induced mild activation of caspases 8 and 9, whereas upon combination treatment, both caspases were strongly activated. In CP70, cisplatin induced upregulation of procaspase 8 levels. Recombinant human TRAIL induced a slight activation of caspases 8 and 9, whereas the combination of cisplatin and rhTRAIL resulted in strong activation of both caspases (Figure 2D).

### Caspase 8 protein levels affect rhTRAIL-induced apoptosis

**Figure 3:**
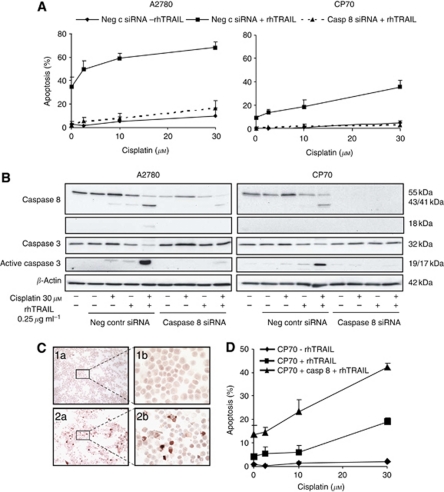
Caspase 8 downregulation inhibits cisplatin and rhTRAIL-induced apoptosis, whereas caspase 8 upregulation augmented apoptosis induction. A2780 and CP70 were transfected with siRNA against caspase 8 or negative control siRNA (**A** and **B**). The CP70 cells were transiently transfected with a caspase 8 construct (**C** and **D**). At 48 h after transfection, cells were treated with 30 *μ*M cisplatin or medium control for 4 h, after which all cells were washed with PBS. Following 16 h of recovery, cells were treated with 0.25 *μ*g ml^−1^ rhTRAIL or medium control for 4 h. (**A**) Apoptosis induction was determined by acridine orange staining. Caspase 8 siRNA strongly reduced apoptosis induction by rhTRAIL and cisplatin in both cell lines. (**B**) Caspase 8 and 3 expression was determined using western blot analysis. The exposure time of the caspase 8 blot of CP70 was increased compared with the blot of A2780. *β*-Actin serves as a loading control. The blots are representative for at least three independent experiments. (**C**) Cytospins of CP70 cells (1,2) were generated and stained for caspase 8 and show increased caspase 8 levels following transfection (2a+b) compared with untransfected cells (1a+b). (**D**) Apoptosis induction following cisplatin and rhTRAIL was determined by acridine orange staining. Caspase 8 upregulation increased apoptosis induction by rhTRAIL alone and by rhTRAIL in combination with cisplatin.

As caspase 8 protein levels are reduced in CP70 compared with A2780, we examined the importance of caspase 8 protein levels for rhTRAIL-sensitivity following cisplatin pre-treatment. Efficient downregulation of caspase 8 with siRNA strongly reduced apoptosis induction following treatment of A2780 and CP70 with rhTRAIL alone and combined with cisplatin (Figure 3A). In caspase 8 siRNA-transfected cells, combined treatment with cisplatin and rhTRAIL minimally induced caspases 8 and 3 activation, whereas in negative control siRNA-transfected cells, strong activation of both caspase 8 and 3 was observed (Figure 3B). It can be noticed that caspase 3 levels are also slightly upregulated following cisplatin in both cell lines (Figure 3B). A role for caspase 3 in rhTRAIL sensitisation can therefore not be excluded. However, the importance of caspase 8 levels was also demonstrated by transient upregulation of caspase 8 in CP70 that enhanced apoptosis induction by rhTRAIL alone and by the rhTRAIL–cisplatin combination (Figures 3C and D).

### Cisplatin increases caspase 8 mRNA levels

**Figure 4:**
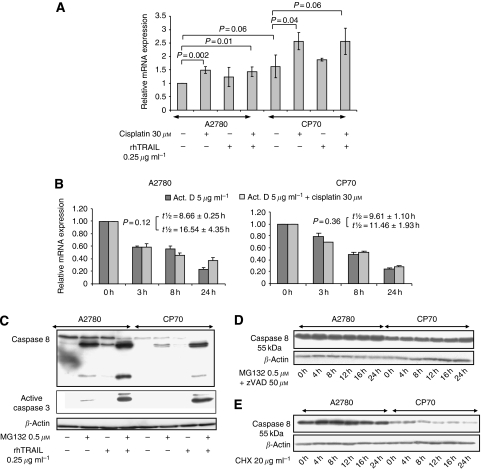
Stability of caspase 8 mRNA or protein does not differ between A2780 and CP70. Differences in caspase 8 protein levels in CP70 are due to changes in protein translation. (**A**) Expression of caspase 8 mRNA was determined with quantitative RT–PCR after exposure to 30 *μ*M cisplatin, 0.25 *μ*g ml^−1^ rhTRAIL or the combination in both cell lines. Cells were incubated for 4 h with cisplatin, after which the cells were washed and total RNA was isolated 20 h after cisplatin exposure. Total RNA was isolated 4 h after exposure to rhTRAIL. Quantitative RT–PCR was carried out on cDNA with the SYBR Green method. Quantification was performed with the standard curve method with GAPDH as reference. Basic mRNA levels between A2780 and CP70 were not different. Cisplatin induced caspase 8 mRNA by 1.5 fold. (**B**) To assess caspase 8 mRNA stability, cells were exposed to 30 *μ*M cisplatin for 4 h or left untreated. Hereafter, all conditions were washed with PBS and received new medium, followed by addition of 5 *μ*g ml^−1^ actinomycin D (Act D). Total RNA was extracted at the time of Act D addition (*t*=0 h) and at the indicated time points. Quantitative RT–PCR was performed as in (**A**). Data represent the mean±s.d. of at least three independent experiments. (**C**) A2780 and CP70 were exposed to 0.5 *μ*M of the proteasome inhibitor MG132 for 24 h. The following day either rhTRAIL was added the last 4 h of incubation, or the cells were left untreated. Then cells were lysed and subjected to western blot analysis with caspase 8 and 3 antibodies. (**D** and **E**) The A2780 and CP70 cell lines were treated with 0.5 *μ*M MG132 for the indicated time points in the presence of 50 *μ*M of z-VAD and lysed or treated with 20 *μ*g ml^−1^ cycloheximide (CHX) for the indicated time points and lysed. Following SDS–PAGE, immunoblotting was carried out with caspase 8 antibodies. *β*-Actin serves as a loading control. All immunoblots are representative for at least three independent experiments.

We investigated the mechanism causing reduced basal caspase 8 protein levels and cisplatin-induced caspase 8 upregulation in CP70. Basal caspase 8 mRNA levels were slightly higher in CP70 than in A2780. Exposure to cisplatin resulted in 1.5-fold induction of caspase 8 mRNA in both the cell lines (Figure 4A). Possible differences in mRNA stability over time and in response to cisplatin were determined using actinomycin D. Caspase 8 mRNA degradation in the absence or presence of cisplatin was not different between A2780 and CP70 (*P*=0.35) (Figure 4B). These results show that cisplatin does not influence caspase 8 mRNA degradation, indicating that the induction of mRNA is caused by increased transcription.

### Caspase 8 protein translation is reduced in CP70

The similar caspase 8 mRNA expression, whereas different basal caspase 8 protein levels were observed, suggest possible changes in caspase 8 protein translation or degradation in CP70. Cells were exposed to the proteasome inhibitor MG132 to test whether increased proteasomal degradation causes the reduced caspase 8 protein levels in CP70. Treatment with MG312 induced upregulation of active caspase 8, leading to concomitant synergy with rhTRAIL (Figure 4C). To prevent caspase 8 activation by MG132 treatment, cells were co-incubated with the caspase inhibitor zVAD for 24 h, which did not lead to a change in full-length caspase 8 levels (Figure 4D). Cycloheximide exposure for up to 24 h slightly affected caspase 8 levels in both cell lines, whereas caspase 8 degradation was not different between A2780 and CP70 (Figure 4E). These results show that not caspase 8 protein stability but rather a decreased translation of caspase 8 mRNA is causing the reduced caspase 8 expression in CP70.

### Increased caspase 8 but not the caspase 8/c-FLIP ratio is involved in the response to cisplatin and rhTRAIL in CP70

**Figure 5:**
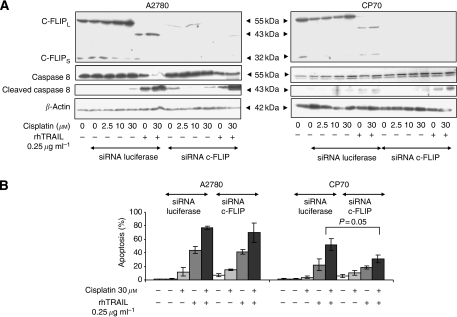
The caspase 8/c-FLIP ratio is not involved in resistance to rhTRAIL. Cells were transfected with siRNA against c-FLIP or luciferase. The next day, cells were exposed to cisplatin for 4 h, after which all cells including those conditions left unexposed were washed. The following day after 4 h incubation with or without 0.25 *μ*g ml^−1^ rhTRAIL cell lysates were made (**A**) Cellular FLICE-inhibitory protein (c-FLIP) and caspase 8 cleavage was determined with western blot analysis. The exposure time of the caspase 8 blot of CP70 was increased compared with the blot of A2780. The blots are representative for at least three independent experiments. (**B**) A small fraction of the same cell suspension used for western blot was plated in a 96-wells plate and apoptosis levels were determined with acridine orange apoptosis assays. Data represent the mean±s.d. of at least three independent experiments. After siRNA against c-FLIP a significant decrease in apoptosis occurred in CP70 after treatment with cisplatin and rhTRAIL (*P*<0.05).

Difference in caspase 8/c-FLIP ratio between A2780 and CP70 might contribute to resistance in CP70. However, efficient downregulation of both c-FLIP_L_ and c-FLIP_s_ with siRNA did not lead to increased apoptosis after either treatment regimen, and did not affect caspase 8 cleavage (Figure 5A). Downregulation even led to a significant decrease in apoptosis in CP70 (Figure 5B). These results indicate that solely the elevated caspase 8 level is involved in the onset of apoptosis after combination treatment in CP70. Moreover, c-FLIP even promotes caspase 8 activation in these cells.

### P53 causes cisplatin-induced DR5 expression, but is not involved in rhTRAIL sensitisation

**Figure 6:**
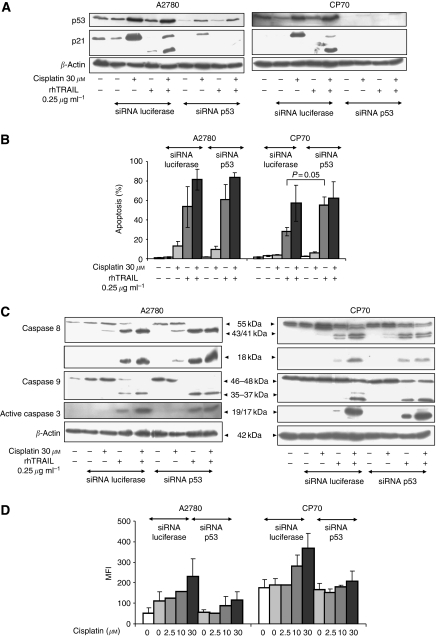
The tumor suppressor protein p53 causes cisplatin-induced DR5 expression but is not required for rhTRAIL sensitisation of A2780 and CP70. The A2780 and CP70 cell lines were transfected with siRNA against p53 or luciferase and were 24 h later exposed to 30 *μ*M of cisplatin during 4 h, after which all cells including those not exposed to cisplatin, were washed. The next day the cells were treated with 0.25 *μ*g ml^−1^ rhTRAIL for 4 h. (**A**) Subsequently, western blotting of p53 and p21 levels was performed on the lysates. *β*-Actin serves as a loading control. (**B**) A small fraction of the same cell suspension used for western blot was plated in a 96-wells plate and apoptosis levels were determined with acridine orange apoptosis assays. After downregulation of p53, a significant increase in apoptosis occurred in CP70 after treatment with rhTRAIL. (**C**) Cleavage patterns of caspase 8, caspase 9 and caspase 3 were determined on lysates after transfection with siRNA against luciferase and p53 as indicated above. The exposure time of the caspase 8 blot of CP70 was increased compared with the blot of A2780. *β*-Actin serves as a loading control. All blots are representative for at least three independent experiments. (**D**) At 24 h after siRNA transfection, the cells were exposed for 4 h to 2.5, 10 and 30 *μ*M cisplatin, after which all cells were washed. The next day cells were collected and analysed for DR5 expression by flow FACS cytometry. DR5 expression is represented as MFI. Data represent the mean±s.d. of at least three independent experiments.

As p53-induced upregulation of DR5 is frequently described to be instrumental in the synergistic effect between chemotherapeutics and rhTRAIL (Wu *et al*, 2004), we asked whether the synergistic effect of cisplatin and rhTRAIL was p53 dependent. The tumor suppressor protein p53 was efficiently and functionally downregulated using siRNA in A2780 and CP70 as shown by the decreased expression of p21 (Waf1/Cip1), a transcriptional target of p53 (Figure 6A). In response to cisplatin, p53 levels rose slightly in p53-suppressed cells, but the levels remained far below those in the untreated luciferase siRNA-transfected cells. Apoptosis induction (Figure 6B) and activation of caspases 8, 9 and 3 (Figure 6C) were not affected by p53 siRNA. Following p53 downregulation, basal DR5 membrane expression maintained unchanged, whereas cisplatin-induced DR5 membrane expression was effectively suppressed in A2780 and CP70 (Figure 6D). These results show that p53-dependent upregulation of DR5 is not involved in the synergy between cisplatin and rhTRAIL. In addition, DISC-IP using a DR5 antibody following cisplatin treatment showed that DISC formation is not impeded in CP70 (data not shown).

## Discussion

In the present study, we show that the cellular caspase 8 protein level is an important determinant of sensitivity to rhTRAIL-induced apoptosis in an isogenic ovarian cancer cell line model of acquired cisplatin resistance. Combination of cisplatin and rhTRAIL effectively induced apoptosis, with cisplatin-induced caspase 8 protein expression being the key factor of sensitisation to rhTRAIL in CP70. The central role of caspase 8 was further indicated by our observation that cisplatin-induced DR5 upregulation was not an important sensitising factor. As caspase 8 mRNA levels do not differ significantly between A2780 and CP70, the low caspase 8 protein levels in CP70 are likely to be caused by decreased translation of caspase 8 protein rather than decreased transcription through methylation or genetic alterations that occur in neuroblastoma (Teitz *et al*, 2000; Takita *et al*, 2001) and in other solid tumours (Kim *et al*, 2003; Soung *et al*, 2005). Increased caspase 8 protein degradation in CP70 was ruled out, as the effect of inhibition of protein synthesis and proteasomal degradation on caspase 8 levels was not different between A2780 and CP70.

Translational and post-translational modifications are important factors of the expression levels and activity of key proteins in the regulation of cell survival and apoptosis such as p53 (Xu, 2003), p73 (Ozaki *et al*, 2005), XIAP and APAF-1 (Holcik and Sonenberg, 2005). Recently, attention has been drawn to microRNAs (miRNAs) as important regulators of translation and mRNA stability (Kent and Mendell, 2006). Deregulation of several miRNAs was described for ovarian cancer, and an important role for miRNAs in cisplatin and TRAIL resistance was shown in ovarian and lung cancer, respectively (Garofalo *et al*, 2008; Yang *et al*, 2008). In melanoma cells, an inducible post-translational modification of mRNA contributed to TRAIL resistance, in which cytosolic proteins could suppress DR5 protein expression by binding to the 3′-untranslated region of DR5-mRNA (Zhang *et al*, 2004). These studies support our hypothesis that a post-transcriptional mechanism is involved in the low expression of caspase 8 in CP70. The fact that most prominently in CP70, caspase 8 translation could be enhanced by cisplatin exposure argues in favour of a reversible block of translation. Low caspase 8 levels were previously shown to contribute to rhTRAIL resistance in several cell line models and tumours (Hopkins-Donaldson *et al*, 2000; Zhang *et al*, 2005). Regarding the role of caspase 8 and the extrinsic apoptotic pathway in chemotherapy-induced cell death, conflicting reports have been published. Death receptor-dependent and -independent activation of caspase 8 was involved in the response to different chemotherapeutic drugs (Fulda *et al*, 2001; Spierings *et al*, 2003; Longley *et al*, 2006). In addition, resistance to these drugs through defects in the extrinsic pathway further supported the involvement of this pathway in chemotherapy-induced cell death (Abedini *et al*, 2004; Longley *et al*, 2006).

Besides the differential expression of caspase 8, we found no other proteins that could account for the difference in rhTRAIL sensitivity between A2780 and CP70. As caspase 3 was slightly upregulated in both cell lines following cisplatin, a role for caspase 3 and hence also for XIAP can not be completely ruled out (Fraser *et al*, 2003). However, basal caspase 3 levels did not differ between the two cell lines, excluding caspase 3 as a determinant for the difference in rhTRAIL sensitivity. A reduced caspase 8/c-FLIP ratio can result in resistance to DR signalling (Mitsiades *et al*, 2002; Baumler *et al*, 2003), whereas an increased caspase 8/c-FLIP ratio in the DISC can cause sensitisation to rhTRAIL by chemotherapeutics (Lacour *et al*, 2003; Ganten *et al*, 2004). In our cell lines, downregulation of c-FLIP did not increase apoptosis induction. In the resistant CP70, downregulation of c-FLIP even resulted in a significant drop in apoptosis levels after exposure to cisplatin and rhTRAIL. This suggests that c-FLIP functions as a pro-apoptotic protein in CP70 and that the caspase 8 level is the most important determinant for rhTRAIL sensitivity. Although extensive literature exists on the anti-apoptotic function of c-FLIP, a role of c-FLIP in activation of caspase 8 was also reported (Micheau *et al*, 2002; Boatright *et al*, 2004). It is postulated that the c-FLIP_L_ concentration at the DISC determines whether caspase 8 activation or inhibition occurs (Boatright *et al*, 2004; Peter, 2004). Thus, it can be reasoned that the caspase 8-activating function of FLIP applies to A2780 and CP70, especially when low caspase 8 levels are present and heterodimerisation can occur more easily than homodimerisation.

Upregulation of DR4 or DR5, which can occur in a p53-dependent or -independent manner in response to chemotherapeutics (Wu *et al*, 1997; Sheikh *et al*, 1998), is often described as a key event in the synergistic effect between chemotherapeutics and rhTRAIL (Wu *et al*, 2004). In this study, we show that the increase of DRs after chemotherapy treatment might just be an epiphenomenon, instead of a key event in modulation of rhTRAIL-induced cell death by chemotherapeutics. Downregulation of p53 did not affect apoptosis induction in both cell lines, whereas the substantial increase of DR5 after treatment with cisplatin was almost completely abrogated by p53 downregulation. It has been shown that p53 is required for cisplatin-induced apoptosis either via p53-dependent ubiquitination of FLIP or through p53-induced XIAP and Akt downregulation when used as a single agent (Fraser *et al*, 2003; Abedini *et al*, 2008). These results may explain why CP70 has reduced caspase 8 levels compared with A2780. However, in our experiments we have chosen to use cisplatin in a short-treatment setting. This was followed by a short treatment with rhTRAIL, and we have demonstrated that upon combination of cisplatin and rhTRAIL, p53 is no longer required for apoptosis induction.

Conclusively, these results show that cisplatin enhances rhTRAIL sensitivity in both cisplatin-sensitive and -resistant cells. Induction of caspase 8 protein expression is the key factor of rhTRAIL sensitisation.

## References

[bib1] Abedini MR, Muller EJ, Brun J, Bergeron R, Gray DA, Tsang BK (2008) Cisplatin induces p53-dependent FLICE-like inhibitory protein ubiquitination in ovarian cancer cells. Cancer Res 68: 4511–4517 18559494 10.1158/0008-5472.CAN-08-0673

[bib2] Abedini MR, Qiu Q, Yan X, Tsang BK (2004) Possible role of FLICE-like inhibitory protein (FLIP) in chemoresistant ovarian cancer cells *in vitro*. Oncogene 23: 6997–7004 15258564 10.1038/sj.onc.1207925

[bib3] Agarwal R, Kaye SB (2003) Ovarian cancer: strategies for overcoming resistance to chemotherapy. Nat Rev Cancer 3: 502–516 12835670 10.1038/nrc1123

[bib4] Ashkenazi A, Holland P, Eckhardt SG (2008) Ligand-based targeting of apoptosis in cancer: the potential of recombinant human apoptosis ligand 2/tumor necrosis factor-related apoptosis-inducing ligand (rhApo2L/TRAIL). J Clin Oncol 26: 3621–3630 18640940 10.1200/JCO.2007.15.7198

[bib5] Ashkenazi A, Pai RC, Fong S, Leung S, Lawrence DA, Marsters SA, Blackie C, Chang L, McMurtrey AE, Hebert A, DeForge L, Koumenis IL, Lewis D, Harris L, Bussiere J, Koeppen H, Shahrokh Z, Schwall RH (1999) Safety and antitumor activity of recombinant soluble Apo2 ligand. J Clin Invest 104: 155–162 10411544 10.1172/JCI6926 PMC408479

[bib6] Baumler C, Duan F, Onel K, Rapaport B, Jhanwar S, Offit K, Elkon KB (2003) Differential recruitment of caspase 8 to cFlip confers sensitivity or resistance to Fas-mediated apoptosis in a subset of familial lymphoma patients. Leuk Res 27: 841–851 12804643 10.1016/s0145-2126(03)00018-3

[bib7] Boatright KM, Deis C, Denault JB, Sutherlin DP, Salvesen GS (2004) Activation of caspases-8 and -10 by FLIP(L). Biochem J 382: 651–657 15209560 10.1042/BJ20040809 PMC1133822

[bib8] Boatright KM, Renatus M, Scott FL, Sperandio S, Shin H, Pedersen IM, Ricci JE, Edris WA, Sutherlin DP, Green DR, Salvesen GS (2003) A unified model for apical caspase activation. Mol Cell 11: 529–541 12620239 10.1016/s1097-2765(03)00051-0

[bib9] Chun HJ, Zheng L, Ahmad M, Wang J, Speirs CK, Siegel RM, Dale JK, Puck J, Davis J, Hall CG, Skoda-Smith S, Atkinson TP, Straus SE, Lenardo MJ (2002) Pleiotropic defects in lymphocyte activation caused by caspase-8 mutations lead to human immunodeficiency. Nature 419: 395–399 12353035 10.1038/nature01063

[bib10] Crijns AP, Boezen HM, Schouten JP, Arts HJ, Hofstra RM, Willemse PH, Vries de EG, Van der Zee AG (2003) Prognostic factors in ovarian cancer: current evidence and future prospects. Eur J Cancer S1: 127–145

[bib11] Duiker EW, de Vries EG, Mahalingam D, Meersma GJ, Boersma-van EW, Hollema H, Lub-de Hooge MN, van Dam GM, Cool RH, Quax WJ, Samali A, van der Zee AG, de Jong S (2009) Enhanced antitumor efficacy of a DR5-specific TRAIL variant over recombinant human TRAIL in a bioluminescent ovarian cancer xenograft model. Clin Cancer Res 15: 2048–2057 19276284 10.1158/1078-0432.CCR-08-1535

[bib12] Duiker EW, van der Zee AG, de Graeff P, Boersma-van Ek W, Hollema H, de Bock GH, de Jong S, de Vries EG (2010) The extrinsic apoptosis pathway and its prognostic impact in ovarian cancer. Gynecol Oncol 116: 549–555 19959214 10.1016/j.ygyno.2009.09.014

[bib13] Elbashir SM, Harborth J, Lendeckel W, Yalcin A, Weber K, Tuschl T (2001) Duplexes of 21-nucleotide RNAs mediate RNA interference in cultured mammalian cells. Nature 411: 494–498 11373684 10.1038/35078107

[bib14] Fraser M, Leung BM, Yan X, Dan HC, Cheng JQ, Tsang BK (2003) p53 is a determinant of X-linked inhibitor of apoptosis protein/Akt-mediated chemoresistance in human ovarian cancer cells. Cancer Res 63: 7081–7088 14612499

[bib15] Fulda S, Meyer E, Friesen C, Susin SA, Kroemer G, Debatin KM (2001) Cell type specific involvement of death receptor and mitochondrial pathways in drug-induced apoptosis. Oncogene 20: 1063–1075 11314043 10.1038/sj.onc.1204141

[bib16] Ganten TM, Haas TL, Sykora J, Stahl H, Sprick MR, Fas SC, Krueger A, Weigand MA, Grosse-Wilde A, Stremmel W, Krammer PH, Walczak H (2004) Enhanced caspase-8 recruitment to and activation at the DISC is critical for sensitisation of human hepatocellular carcinoma cells to TRAIL-induced apoptosis by chemotherapeutic drugs. Cell Death Differ 11(Suppl 1): S86–S96 15105837 10.1038/sj.cdd.4401437

[bib17] Garofalo M, Quintavalle C, Di LG, Zanca C, Romano G, Taccioli C, Liu CG, Croce CM, Condorelli G (2008) MicroRNA signatures of TRAIL resistance in human non-small cell lung cancer. Oncogene 27: 3845–3855 18246122 10.1038/onc.2008.6

[bib18] Godwin AK, Meister A, O′Dwyer PJ, Huang CS, Hamilton TC, Anderson ME (1992) High resistance to cisplatin in human ovarian cancer cell lines is associated with marked increase of glutathione synthesis. Proc Natl Acad Sci USA 89: 3070–3074 1348364 10.1073/pnas.89.7.3070 PMC48805

[bib19] Herbst RS, Eckhardt SG, Kurzrock R, Ebbinghaus S, O'Dwyer PJ, Gordon MS, Novotny W, Goldwasser MA, Tohnya TM, Lum BL, Ashkenazi A, Jubb AM, Mendelson DS (2010) Phase I dose-escalation study of recombinant human Apo2L/TRAIL, a dual proapoptotic receptor agonist, in patients with advanced cancer. J Clin Oncol 28: 2839–2846 20458040 10.1200/JCO.2009.25.1991

[bib20] Holcik M, Sonenberg N (2005) Translational control in stress and apoptosis. Nat Rev Mol Cell Biol 6: 318–327 15803138 10.1038/nrm1618

[bib21] Hopkins-Donaldson S, Bodmer JL, Bourloud KB, Brognara CB, Tschopp J, Gross N (2000) Loss of caspase-8 expression in highly malignant human neuroblastoma cells correlates with resistance to tumor necrosis factor-related apoptosis-inducing ligand-induced apoptosis. Cancer Res 60: 4315–4319 10969767

[bib22] Johnson SW, Perez RP, Godwin AK, Yeung AT, Handel LM, Ozols RF, Hamilton TC (1994) Role of platinum-DNA adduct formation and removal in cisplatin resistance in human ovarian cancer cell lines. Biochem Pharmacol 47: 689–697 8129746 10.1016/0006-2952(94)90132-5

[bib23] Kent OA, Mendell JT (2006) A small piece in the cancer puzzle: microRNAs as tumor suppressors and oncogenes. Oncogene 25: 6188–6196 17028598 10.1038/sj.onc.1209913

[bib24] Kim HS, Lee JW, Soung YH, Park WS, Kim SY, Lee JH, Park JY, Cho YG, Kim CJ, Jeong SW, Nam SW, Kim SH, Lee JY, Yoo NJ, Lee SH (2003) Inactivating mutations of caspase-8 gene in colorectal carcinomas. Gastroenterology 125: 708–715 12949717 10.1016/s0016-5085(03)01059-x

[bib25] Lacour S, Micheau O, Hammann A, Drouineaud V, Tschopp J, Solary E, manche-Boitrel MT (2003) Chemotherapy enhances TNF-related apoptosis-inducing ligand DISC assembly in HT29 human colon cancer cells. Oncogene 22: 1807–1816 12660816 10.1038/sj.onc.1206127

[bib26] Li H, Zhu H, Xu CJ, Yuan J (1998) Cleavage of BID by caspase 8 mediates the mitochondrial damage in the Fas pathway of apoptosis. Cell 94: 491–501 9727492 10.1016/s0092-8674(00)81590-1

[bib27] Longley DB, Wilson TR, McEwan M, Allen WL, McDermott U, Galligan L, Johnston PG (2006) c-FLIP inhibits chemotherapy-induced colorectal cancer cell death. Oncogene 25: 838–848 16247474 10.1038/sj.onc.1209122

[bib28] Mahalingam D, Szegezdi E, Keane M, de Jong S, Samali A (2009) TRAIL receptor signalling and modulation: are we on the right TRAIL? Cancer Treat Rev 35: 280–288 19117685 10.1016/j.ctrv.2008.11.006

[bib29] Martinez LA, Naguibneva I, Lehrmann H, Vervisch A, Tchenio T, Lozano G, Harel-Bellan A (2002) Synthetic small inhibiting RNAs: efficient tools to inactivate oncogenic mutations and restore p53 pathways. Proc Natl Acad Sci USA 99: 14849–14854 12403821 10.1073/pnas.222406899 PMC137507

[bib30] Micheau O, Thome M, Schneider P, Holler N, Tschopp J, Nicholson DW, Briand C, Grutter MG (2002) The long form of FLIP is an activator of caspase-8 at the Fas death-inducing signaling complex. J Biol Chem 277: 45162–45171 12215447 10.1074/jbc.M206882200

[bib31] Mitsiades N, Mitsiades CS, Poulaki V, Anderson KC, Treon SP (2002) Intracellular regulation of tumor necrosis factor-related apoptosis-inducing ligand-induced apoptosis in human multiple myeloma cells. Blood 99: 2162–2171 11877293 10.1182/blood.v99.6.2162

[bib32] Ozaki T, Hosoda M, Miyazaki K, Hayashi S, Watanabe K, Nakagawa T, Nakagawara A (2005) Functional implication of p73 protein stability in neuronal cell survival and death. Cancer Lett 228: 29–35 15907364 10.1016/j.canlet.2004.12.050

[bib33] Peter ME (2000) The TRAIL DISCussion: it is FADD and caspase-8!. Cell Death Differ 7: 759–760 11042670 10.1038/sj.cdd.4400735

[bib34] Peter ME (2004) The flip side of FLIP. Biochem J 382: e1–e3 15317488 10.1042/BJ20041143 PMC1133836

[bib35] Pitti RM, Marsters SA, Ruppert S, Donahue CJ, Moore A, Ashkenazi A (1996) Induction of apoptosis by Apo-2 ligand, a new member of the tumor necrosis factor cytokine family. J Biol Chem 271: 12687–12690 8663110 10.1074/jbc.271.22.12687

[bib36] Sheikh MS, Burns TF, Huang Y, Wu GS, Amundson S, Brooks KS, Fornace Jr AJ, El-Deiry WS (1998) p53-dependent and -independent regulation of the death receptor KILLER/DR5 gene expression in response to genotoxic stress and tumor necrosis factor alpha. Cancer Res 58: 1593–1598 9563466

[bib37] Soung YH, Lee JW, Kim SY, Sung YJ, Park WS, Nam SW, Kim SH, Lee JY, Yoo NJ, Lee SH (2005) Caspase-8 gene is frequently inactivated by the frameshift somatic mutation 1225_1226delTG in hepatocellular carcinomas. Oncogene 24: 141–147 15531912 10.1038/sj.onc.1208244

[bib38] Spierings DC, de Vries EG, Vellenga E, de Jong S (2003) Loss of drug-induced activation of the CD95 apoptotic pathway in a cisplatin-resistant testicular germ cell tumor cell line. Cell Death Differ 10: 808–822 12815464 10.1038/sj.cdd.4401248

[bib39] Takita J, Yang HW, Chen YY, Hanada R, Yamamoto K, Teitz T, Kidd V, Hayashi Y (2001) Allelic imbalance on chromosome 2q and alterations of the caspase 8 gene in neuroblastoma. Oncogene 20: 4424–4432 11466626 10.1038/sj.onc.1204521

[bib40] Teitz T, Wei T, Valentine MB, Vanin EF, Grenet J, Valentine VA, Behm FG, Look AT, Lahti JM, Kidd VJ (2000) Caspase 8 is deleted or silenced preferentially in childhood neuroblastomas with amplification of MYCN. Nat Med 6: 529–535 10802708 10.1038/75007

[bib41] Wernyj RP, Morin PJ (2004) Molecular mechanisms of platinum resistance: still searching for the Achilles’ heel. Drug Resist Updat 7: 227–232 15533760 10.1016/j.drup.2004.08.002

[bib42] Wiley SR, Schooley K, Smolak PJ, Din WS, Huang CP, Nicholl JK, Sutherland GR, Smith TD, Rauch C, Smith CA (1995) Identification and characterization of a new member of the TNF family that induces apoptosis. Immunity 3: 673–682 8777713 10.1016/1074-7613(95)90057-8

[bib43] Wu GS, Burns TF, McDonald III ER, Jiang W, Meng R, Krantz ID, Kao G, Gan DD, Zhou JY, Muschel R, Hamilton SR, Spinner NB, Markowitz S, Wu G, El-Deiry WS (1997) KILLER/DR5 is a DNA damage-inducible p53-regulated death receptor gene. Nat Genet 17: 141–143 9326928 10.1038/ng1097-141

[bib44] Wu XX, Ogawa O, Kakehi Y (2004) TRAIL and chemotherapeutic drugs in cancer therapy. Vitam Horm 67: 365–383 15110186 10.1016/S0083-6729(04)67019-1

[bib45] Xu Y (2003) Regulation of p53 responses by post-translational modifications. Cell Death Differ 10: 400–403 12719715 10.1038/sj.cdd.4401182

[bib46] Yang H, Kong W, He L, Zhao JJ, O′Donnell JD, Wang J, Wenham RM, Coppola D, Kruk PA, Nicosia SV, Cheng JQ (2008) MicroRNA expression profiling in human ovarian cancer: miR-214 induces cell survival and cisplatin resistance by targeting PTEN. Cancer Res 68: 425–433 18199536 10.1158/0008-5472.CAN-07-2488

[bib47] Zhang L, Zhu H, Teraishi F, Davis JJ, Guo W, Fan Z, Fang B (2005) Accelerated degradation of caspase-8 protein correlates with TRAIL resistance in a DLD1 human colon cancer cell line. Neoplasia 7: 594–602 16036110 10.1593/neo.04688 PMC1501285

[bib48] Zhang XY, Zhang XD, Borrow JM, Nguyen T, Hersey P (2004) Translational control of tumor necrosis factor-related apoptosis-inducing ligand death receptor expression in melanoma cells. J Biol Chem 279: 10606–10614 14688276 10.1074/jbc.M308211200

